# Development and performance evaluation of fully automated deep learning-based models for myocardial segmentation on T1 mapping MRI data

**DOI:** 10.1038/s41598-024-69529-7

**Published:** 2024-08-14

**Authors:** Mathias Manzke, Simon Iseke, Benjamin Böttcher, Ann-Christin Klemenz, Marc-André Weber, Felix G. Meinel

**Affiliations:** Institute of Diagnostic and Interventional Radiology, Pediatric Radiology and Neuroradiology, University Medical Centre Rostock, Ernst-Heydemann-Str. 6, 18057 Rostock, Germany

**Keywords:** Deep learning, U-Net, Mapping, Cardiac magnetic resonance imaging, Long axis, Short axis, Cardiology, Computational models, Image processing, Machine learning, Predictive medicine

## Abstract

To develop a deep learning-based model capable of segmenting the left ventricular (LV) myocardium on native T1 maps from cardiac MRI in both long-axis and short-axis orientations. Models were trained on native myocardial T1 maps from 50 healthy volunteers and 75 patients using manual segmentation as the reference standard. Based on a U-Net architecture, we systematically optimized the model design using two different training metrics (Sørensen-Dice coefficient = DSC and Intersection-over-Union = IOU), two different activation functions (ReLU and LeakyReLU) and various numbers of training epochs. Training with DSC metric and a ReLU activation function over 35 epochs achieved the highest overall performance (mean error in T1 10.6 ± 17.9 ms, mean DSC 0.88 ± 0.07). Limits of agreement between model results and ground truth were from -35.5 to + 36.1 ms. This was superior to the agreement between two human raters (-34.7 to + 59.1 ms). Segmentation was as accurate for long-axis views (mean error T1: 6.77 ± 8.3 ms, mean DSC: 0.89 ± 0.03) as for short-axis images (mean error ΔT1: 11.6 ± 19.7 ms, mean DSC: 0.88 ± 0.08). Fully automated segmentation and quantitative analysis of native myocardial T1 maps is possible in both long-axis and short-axis orientations with very high accuracy.

## Introduction

Cardiovascular diseases account for approximately 32% of all deaths worldwide, making them the leading cause of death worldwide^1^. Cardiac magnetic resonance imaging (MRI) has provided various tools for pathology detection for many years and is routinely used to assess the structure and function of the cardiovascular system. In cardiac MRI, mapping sequences are used for quantitative tissue characterization of the myocardium^2^. The myocardial T1 relaxation reflects biophysical properties of myocardial muscle cells and the surrounding extracellular space. Altered myocardial T1 relaxation times typically result from fibrosis, inflammation, edema or storage disease^3^. Quantification of T1 relaxation times is therefore helpful to diagnose pathologies affecting the myocardium.

In clinical practice, cardiovascular imaging specialists often draw regions of interest (ROI) within the T1 maps manually to obtain T1 relaxation times. This process is time-consuming, tedious and prone to variation between readers. Therefore, fully automated contouring of mapping data would be beneficial to efficiently and objectively quantify T1 relaxation times.

There is extensive literature on automated segmentation of cardiac cine sequences for volumetric analysis^4–12^. Only few publications have addressed the challenge of automated segmentation of mapping data^13–19^. All of these studies have been limited to T1 maps acquired in the short-axis (SAX) plane perpendicular to the long axis of the left ventricle and have not investigated whether this is also feasible for long-axis (LAX) images. Thus, there is a paucity of evidence for fully automated segmentation of T1 mapping evidence and a lack of data on the successful development of neural networks for segmenting both SAX and LAX T1 maps with high accuracy.

Therefore, the purpose of this study was to develop and validate a deep learning-based model capable of segmenting the left ventricular myocardium on native T1 maps from cardiac MRI both in LAX and SAX orientation.

## Material and methods

**Figure 1:**
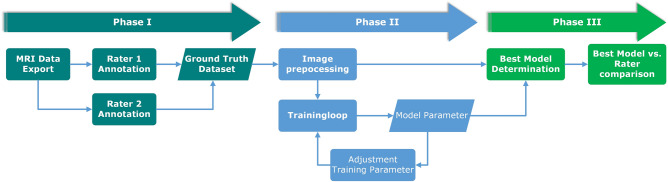
Entire study design flowchart divided in three phases: Phase I (left) contains the data inclusion criteria definition process as well as the data collection and annotation procedure performed by two independent raters who created the ground truth data set; Phase II (center) contains image preprocessing and file operations as well as model specifications and the training loops; During Phase III (right) all post-processing procedures and final analysis were evaluated.

The study was conducted in three phases. In the first phase, the inclusion criteria for data collection were defined and the data was exported from the clinical systems. In the second phase, four different models based on a U-Net architecture were trained over several training epochs. In the third phase, the results of all models were evaluated using a test data set and compared with the ground truth data set of both evaluators. An overview of the overall study design can be seen in Fig. 1.

### Patient selection and study design

**Figure 2:**
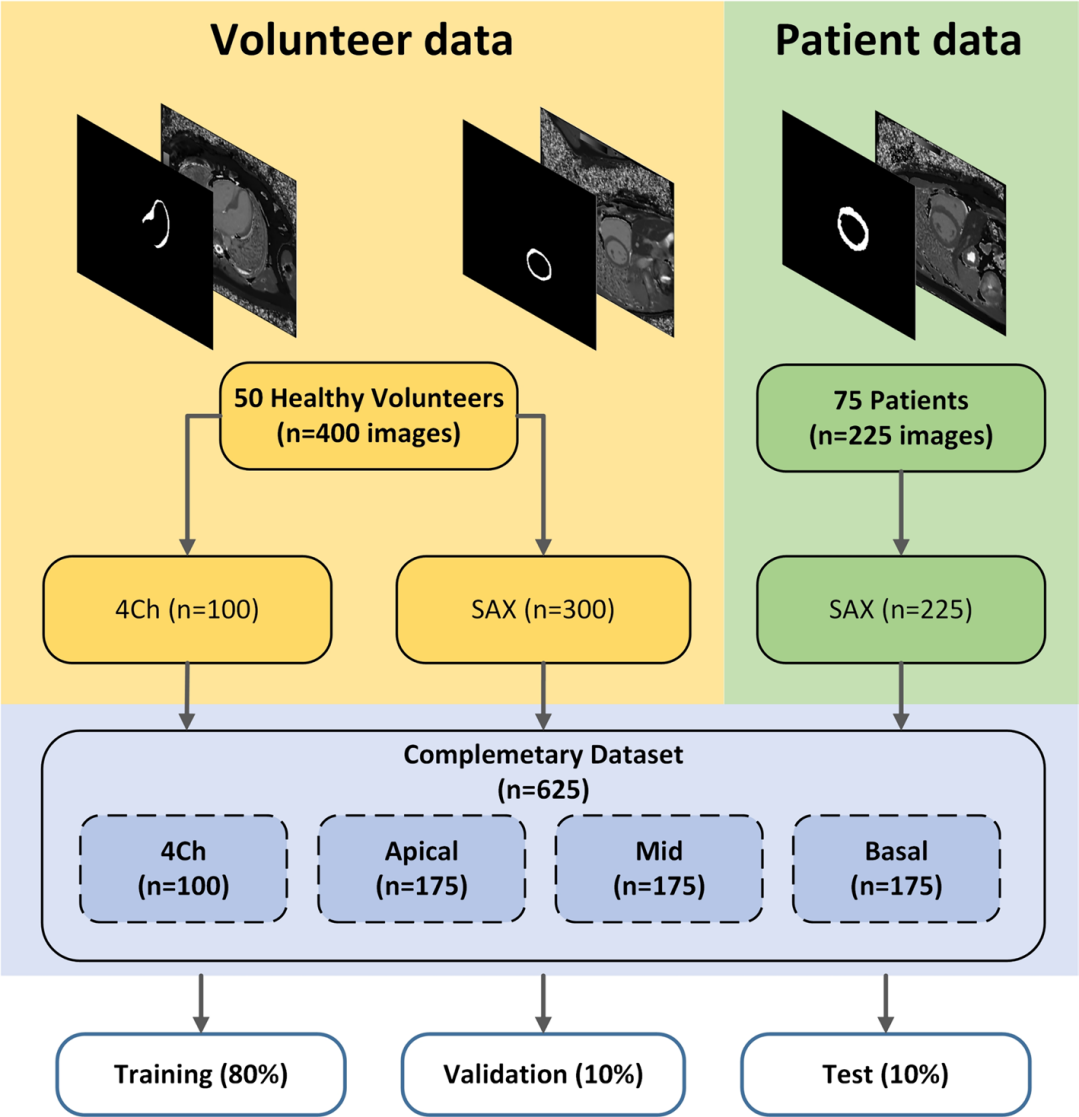
Description of complementary dataset: The visualization shows how the two independently assessed datasets of healthy subjects (n = 50) and patients (n = 75) from routine care were assembled, how many views exist and on which partitions the dataset is distributed.

We included 50 healthy participants whose imaging data from a previous prospective volunteer study^20^ were used and 75 patients who underwent a clinically indicated CMR at 1.5 T between 2020 and 2022 at our institution. All MRI examinations were performed on a 1.5 T MRI scanner (Avanto^fit^ Siemens Healthineers, Erlangen, Germany). Details of imaging protocols and the process of creating the T1 maps have been published previously^20^. Patients were retrospectively selected by searching our institution’s radiology information system. Patients were excluded for the following reasons: severe image artifacts (n = 12), children (n = 3), number of short-axis T1 maps ≠ three (n = 4) and data set errors (n = 4). Figure 2 shows the data collection process.

In the first step, MRI cine images were collected from the 50 healthy volunteers in two rounds and the native T1 maps generated by the scanner were exported. The volunteer dataset consisted of 300 SAX and 100 4Ch image data. In the second step, data from another 75 patients (225 SAX images) were extracted from the clinical systems and segmented by two assessors R1 and R2. In addition, a subset of the patient data-set, with 30 patients (n = 90 images), was segmented by both assessors to evaluate interrater variability.

The Table 1 below shows how each view is distributed across the training, validation, and test data partitions.

**Table 1:** Data partitions overview.

Cohort\View	All	4Ch	API	MID	BAS
Training	500 (80%)	79 (12.64%)	138 (22.08%)	146 (23.36%)	137 (21.92%)
Validation	62 (10%)	8 (1.28%)	17 (2.72%)	14 (2.24%)	23 (3.68%)
Test	63 (10%)	13 (2.08%)	20 (3.20%)	15 (2.40%)	15 (2.40%)

The complementary dataset collected and annotated by two raters is divided in a training, a validation and a test dataset where each subset contains four different views (four chamber view, apical, mid-ventricular, and basal).

### Ethical approval and informed consent

This study was approved by the institutional review board (Ethics Committee, University Medical Center Rostock) and written informed consent was obtained from all 50 healthy volunteers free from cardiovascular disease prior to enrollment. For the additional 75 patients the requirement for informed consent was waived due to the retrospective nature of this part of the investigation. The study conformed to the ethical guidelines of the Helsinki Declaration (revised version from 2013).

### Manual Image segmentation

Both data sets, volunteer data and patient data, were annotated independently by two experienced assessors. Manual segmentation was performed using an open source software solution (ITK-Snap, version 4.0.0).

### Image preprocessing

We selected a U-Net architecture to automatically segment the image data. To achieve good results with smaller training data sets, U-Nets can be effectively trained in combination with data augmentation techniques such as mirroring, rotation, and scaling^21^. These properties are particularly helpful because in the first preprocessing step before training the resolution of the existing MRI images was scaled from (256 × 218, pixel size 1.41 × 1.41 mm) to a uniform format (512 × 512, pixel size 0.70 × 0.60 mm). For practical implementation, scaling makes sense in order to convert the data into a uniform input format and thus be independent of image sizes and pixel spacing. We initially considered performing image padding to avoid warping and maintain square pixels. However, this reduced segmentation accuracy and was therefore not implemented.

To ensure that the residual interpolation error for each individual segmentation mask remained as small as possible, we evaluated various scaling methods (bilinear, Lanczos, Bicubic, pixel-area relation, and nearest neighbor) and determined the influence on the error. For this purpose, mean T1 times were compared between original images (resolution 256 × 218 pixels) and scaled images (resolution 515 × 512). Nearest neighbor interpolation was found to introduce minimal error. No additional data augmentation techniques were used to increase the data volume. Grey-scale images were used without intensity normalization.

### Network architecture

Convolutional layers were used in both the encoder and decoder parts, followed by Rectified Linear Unit (*ReLU*) or *LeakyReLU* activation functions. The encoder sublayers were followed by the *MaxPool* layer to extract global features from the input images, which led to loss of spatial information. In the decoder part, the convolutions were transposed to reconstruct the spatial resolution step by step and create detailed segmentation maps by transforming the activations in higher layers back to the original input size. Skip connections were inserted between the encoder and the decoder and were used to pass features from the encoder to the decoder. This is useful for combining both local and global information and obtaining fine details. The final layer had only one output channel to which the sigmoid activation function was applied to generate the pixel class probabilities, allowing the creation of binary segmentation maps.

The effects of two activation functions were compared during model performance evaluation, as proposed in a previous work^22^.

Therefore, the model was evaluated separately using each of these functions in image segmentation. The activation functions were implemented in the decoder block of the model. For the standard activation function *ReLU*, the output of the neuron corresponds to the level of activation if it is in the positive value range. The output of a neuron can therefore increase arbitrarily with increasing activation. If activations x < 0 the neuron remains deactivated. An updated version of *ReLU* was also used, the *LeakyReLU*. It is one of its upgrades and passes information with a scaling factor α = 0.1.

### Training

To train the deep learning model, cardiac T1 maps of the left ventricle in short-axis view (SAX) and four-chamber view (4Ch) with the corresponding segmentation masks were used as input. The complementary data set, consisting of 625 DICOM images, was randomly divided into three independent subsets: training, validation, and testing data with, 80% of the data set defined for training, 10% of the data set defined for interim validation (during training), and the remaining 10% of the data used for testing. Model training was performed on a Workstation (CPU i7-12,700 2.10 GHz / 32 GB RAM; NVIDIA RTX A2000 / 6 GB GRAM) with CUDA 11.6, Python Tensorflow 2.12.0, Numpy 1.23.5, PyDICOM 2.4.2 and Medpy 0.3.0.

During the training loop, the performance of each model was evaluated using binary accuracy (pixel-wise accuracy) and a metric (DSC and IOU). In particular, two metrics $DSC\left({\text{S}}_{1},{\text{S}}_{2}\right)=2\left|{\text{S}}_{1}\cap {\text{S}}_{2}\right|/\left|{\text{S}}_{1}\right|+\left|{\text{S}}_{2}\right|$ and $IOU\left({\mathbf{S}}_{1},{\mathbf{S}}_{2}\right)=\left|{\text{S}}_{1}\cap {\text{S}}_{2}\right|/|{\text{S}}_{1}\cup {\text{S}}_{2}|$ have been proposed in the relevant literature^22–24^ to measure the congruence of two pixel sets ${\text{S}}_{1}$ and ${\text{S}}_{2}$^25^.

The training data set was divided into batches of 16. A training loop of 5 to 70 training epochs was implemented in steps of 5 epochs using the ADAM optimizer^26^ in combination with variable activation layers and a decreasing learning rate.

### Testing and performance evaluation

The mean T1 times of the ROIs for all trained models were calculated using the test data set, consisting of 63 images in different views (4Ch and SAX). This dataset can be viewed as an external validation dataset in previously unseen images. In addition, the absolute differences in T1 (ΔT1) between the predicted segmentations and the ground truth segmentations as well as the metrics DSC and IOU were determined.

The best model was considered as the model minimizing the error in T1 time quantification ($\overline{\Delta \text{T}1}$) and maximizing both metrics of segmentation accuracy ($\overline{\text{DSC}}$ and $\overline{\text{IOU}}$). For this purpose

a function $D(\Delta \text{T}1,\text{ DSC},\text{ IOU})$ is defined in Eq. (1), which was calculated for all 56 models and all 63 images from the test data set. The arithmetic averages of $\overline{\Delta \text{T}1}$, $\overline{\text{DSC}}$ and $\overline{\text{IOU}}$ were calculated over all 63 absolute values for each model. Because $\overline{\Delta \text{T}1}$ should be as small as possible and $\overline{\text{DSC}}$ and $\overline{\text{IOU}}$ as large as possible, the smallest ${\overline{\Delta \text{T}1} }_{j}$ average and the largest of ${\overline{\text{DSC}} }_{j}$ and ${\overline{\text{IOU}} }_{j}$ were then sought and the minimum of the function $D$ was determined using the least squares method.

We determined the best fitting model as the one minimizing the function ${D}_{j}$ with the definition

1 $${D}_{j}^{2}:= {\left({\overline{\Delta \text{T}1} }_{j}-\text{min}\left({\overline{\Delta \text{T}1} }_{j}\right)\right)}^{2}+ {\left({\overline{\text{DSC}} }_{j}-\text{max}\left({\overline{\text{DSC}} }_{j}\right)\right)}^{2}+ {\left({\overline{\text{IOU}} }_{j}-\text{max}({\overline{\text{IOU}} }_{j})\right)}^{2},$$

where $\text{j}=1,\dots ,56$ is the model number.

**Figure 3:**
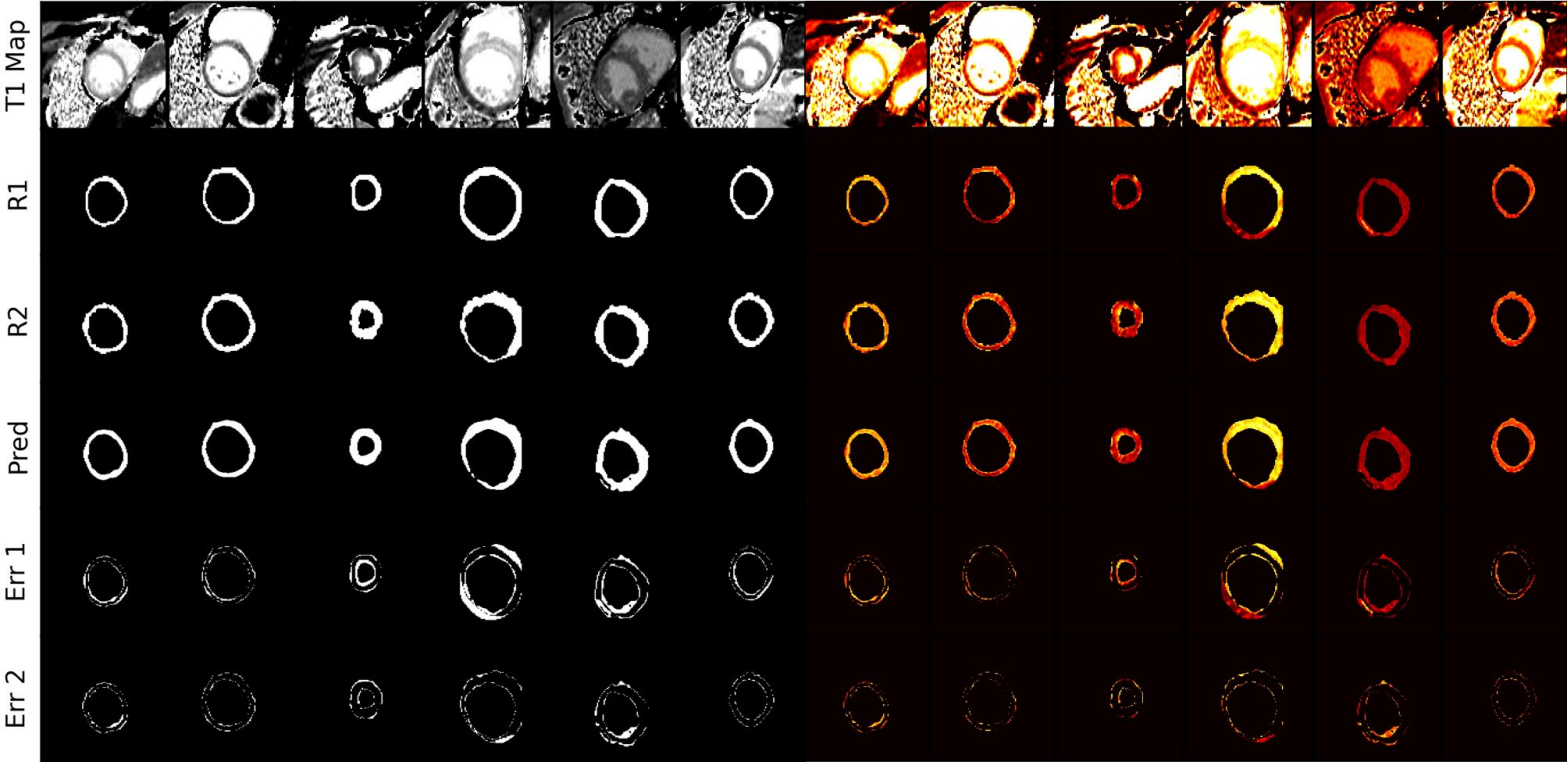
SAX ROI comparison between both Raters and Prediction: In the comparison between original T1 map both raters R1 and R2 and the predicted mask show some slight differences in lower rows Err1 (Prediction-R1 reference) and Err2 (Prediction-R2 reference), Binary masks (left) and intensity masks (right) with a zoom factor of 2.3.

In the second step, the identified best model was compared with the ground truth segmentations of the first rater (R1) and the second rater (R2) as well as the interrater variability, as shown in Fig. 3.

### Statistical analysis

Geometrical segmentation accuracy was evaluated by DSC (Sørensen-Dice-Coefficient) and IOU (Intersection over union). Error in quantitative mapping results was quantified as the error in T1 relaxation times (ΔT1). Bland–Altman analysis was performed to obtain limits of agreement and mean bias between model results. In order to compare model performance and inter-rater variability for the best model, differences in ΔT1, DSC, and IOU were compared between model vs. human and human vs. human (interrater variability) using the Mann–Whitney U test. A value of p < 0.05 was considered statistically significant. Statistical analyzes were performed using the Python (3.11.2) libraries Scipy 1.10.1 and Seaborn 0.12.2

## Results

### Patient characteristics and CMR indications

Our cohort included 50 healthy volunteers (29 female, 21 male, mean age 39.4 ± 13.7 years) and 75 patients (29 female, 46 male, mean age 39.4 ± 13.7 years). The most common CMR indications in patients were known or suspected myocarditis, cardiomyopathy, and ischemic heart disease with 26 (34.7%), 15 (20.0%) and 12 (16.0%) patients, respectively.

### Selection of the optimal model based on overall model performance

**Figure 4:**
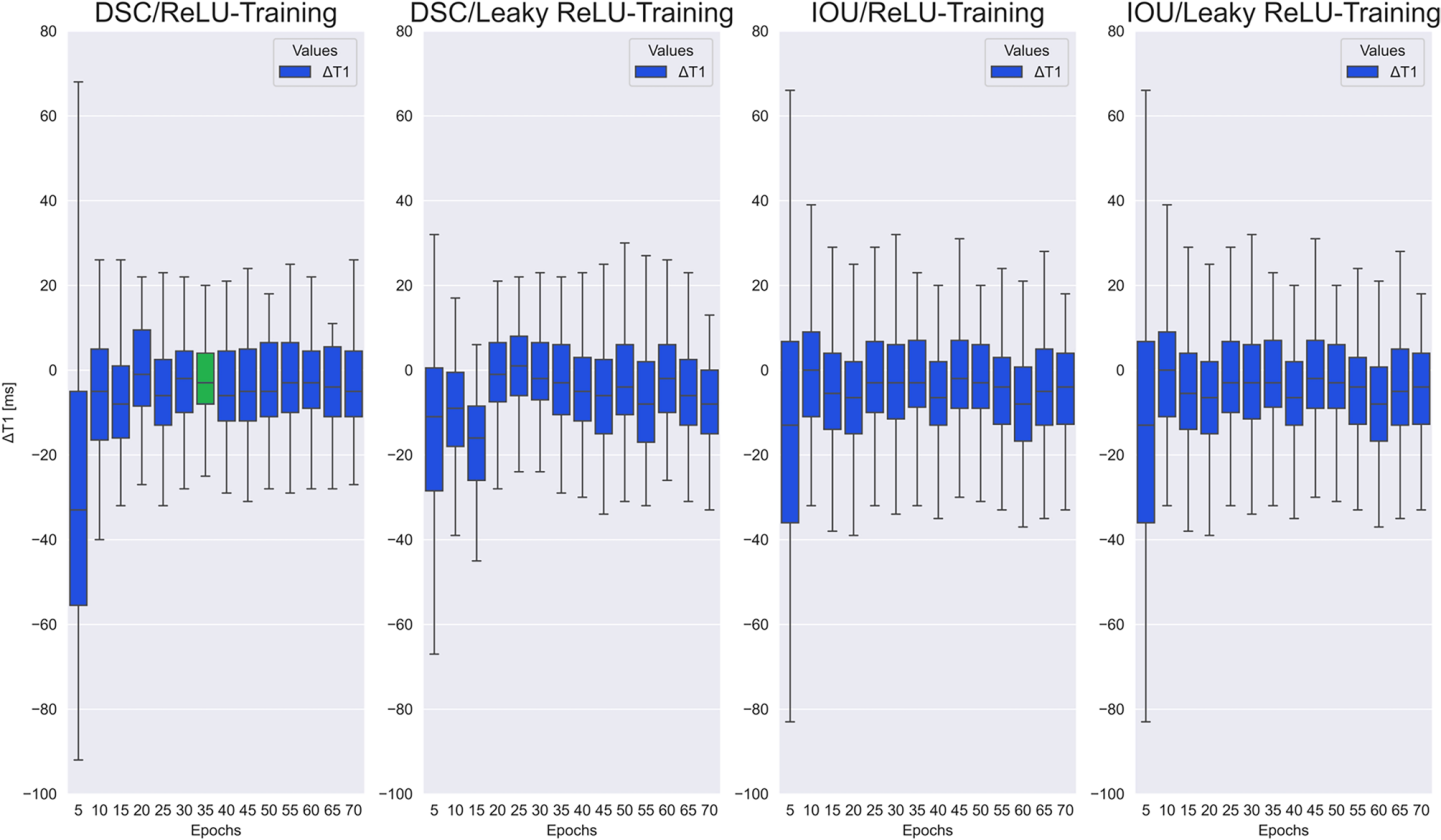
Test cohort model performance with ΔT1: Performance measurement comparison for all 56 trained models with two activation functions (ReLU and Leaky ReLU) and two metrics (DSC and IOU) over all views (SAX and 4Ch) regarding the difference of the averaged ROI relaxation times ΔT1 between prediction and ground truth test data set.

**Figure 5:**
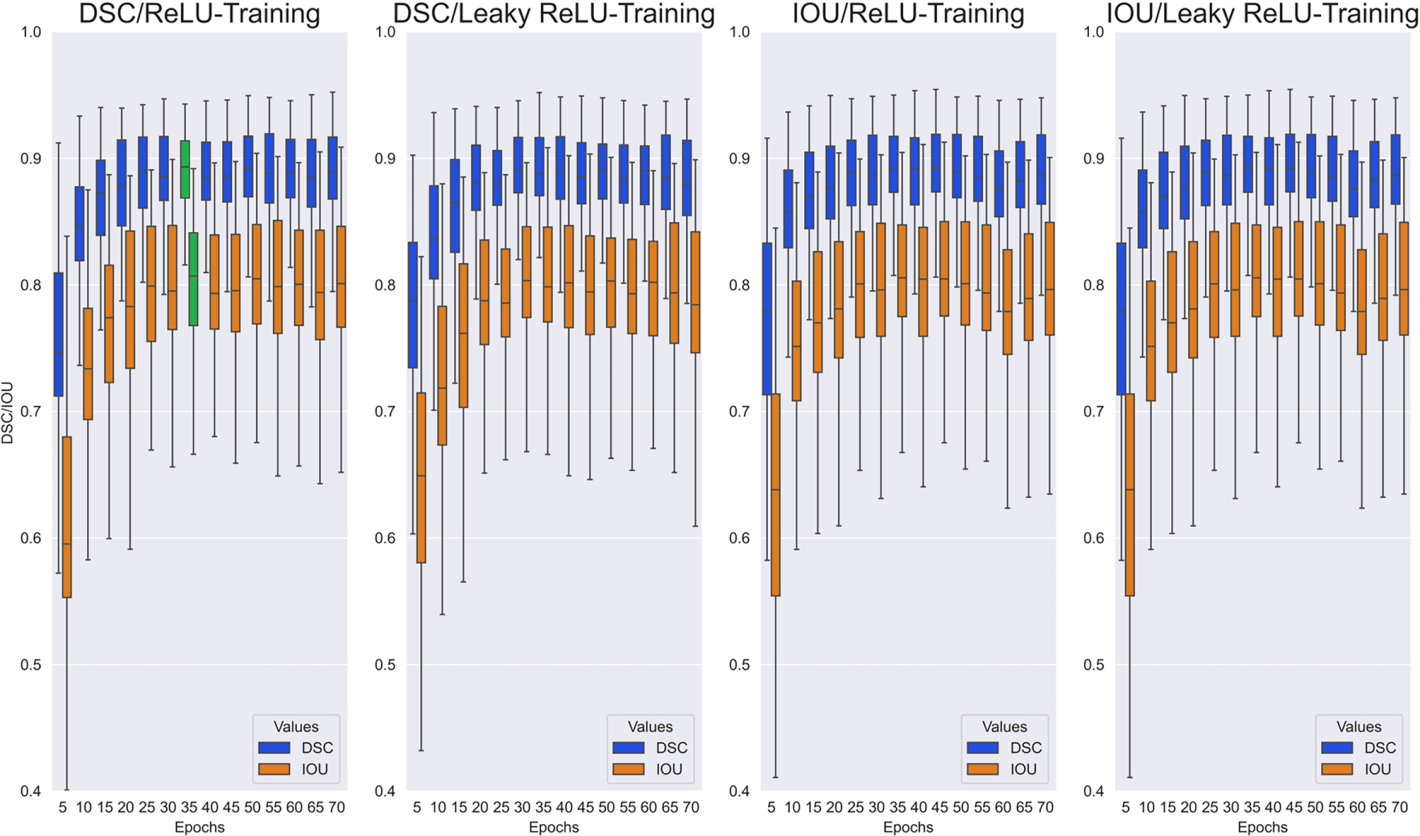
Test cohort model performance with Metrics (DSC/IOU): Performance measurement comparison for all 56 trained models with two activation functions (ReLU and Leaky ReLU) and two metrics (DSC and IOU) and over all views (SAX and 4Ch) regarding the segmentation overlapping between prediction and ground truth test data set also measured with DSC and IOU.

As a result of the image preprocessing, the maximum absolute interpolation error ΔT1 for the scaling process from (256 × 218) to (512 × 512) resolution was ΔT1 =  ± 4 ms, which was considered clinically irrelevant considering that T1 values typically range from 900 to 1200 ms and the limits of agreement for test–retest-reproducibility of T1 mapping are approximately -39 to + 34 ms ^20^. As detailed in the methods section above, the selection of the optimal model was based on a comprehensive strategy minimizing error for the quantitative T1 results (ΔT1) and optimize the segmentation accuracy (DSC, IOU). All three parameters were weighted equally. Figure 4 and Fig. 5 show the overall model performance for all four neural networks as a function of training epochs. A convergence of performance metrics over the number of training epochs is seen, particularly with regards to segmentation accuracy (Fig. 5) with differences becoming small between 25 and 55 training epochs.

Based on Eqs. (1)-, [EP35DSCRE] was determined to be the model with optimal overall performance based on ΔT1, DSC, and IOU (Table 2).

**Table 2:** Model comparison for all views.

Model	$\overline{\Delta T1 }$	$\overline{\text{DSC} }$	$\overline{\text{IOU} }$	$D$
EP35DSCRE	**10.571 (17.891)**	0.879 (0.069)	0.790 (0.088)	**0.013**
EP50IOULR	10.714 (15.839)	0.885 (0.049)	0.797 (0.071)	0.143
EP55IOURE	10.746 (14.612)	0.877 (0.080)	0.787 (0.096)	0.175
EP45IOURE	11.063 (18.483)	**0.887 (0.061)**	**0.801 (0.082)**	0.492
EP25IOURE	11.095 (18.278)	0.881 (0.064)	0.791 (0.083)	0.524
EP50IOURE	11.222 (17.111)	0.881 (0.073)	0.794 (0.091)	0.651
EP45IOULR	11.302 (17.875)	0.884 (0.060)	0.796 (0.080)	0.730
EP70DSCRE	11.317 (15.861)	0.881 (0.073)	0.793 (0.091)	0.746
EP35IOULR	11.333 (19.683)	0.885 (0.058)	0.798 (0.080)	0.762
EP55DSCRE	11.413 (16.531)	0.879 (0.073)	0.789 (0.092)	0.841

Significant values are in bold.

The table shows the aggregated metrics and relaxation times for the ten best models for all of the 63 testing images in all views (4Ch and SAX). In this context, best means a minimal ($\overline{\Delta \text{T}1 })$ and maximum metrics.

The chosen model is marked in green in Fig. 4 and Fig. 5. For this model, mean error in T1 relaxation time was 10.6 ± 17.9 ms, mean DSC was 0.88 ± 0.07, and mean IOU was 0.79 ± 0.09. Specifically, this model shows very high agreement for T1 results with a narrow interquartile range (IQR) of ΔT1 from -7 ms to 5 ms and an overall range of ΔT1 from -25 ms to + 23 ms (Fig. 4). Therefore, the [EP35DSCRE] model was selected for detailed performance evaluation.

### Detailed performance evaluation for the optimal model

**Figure 6:**
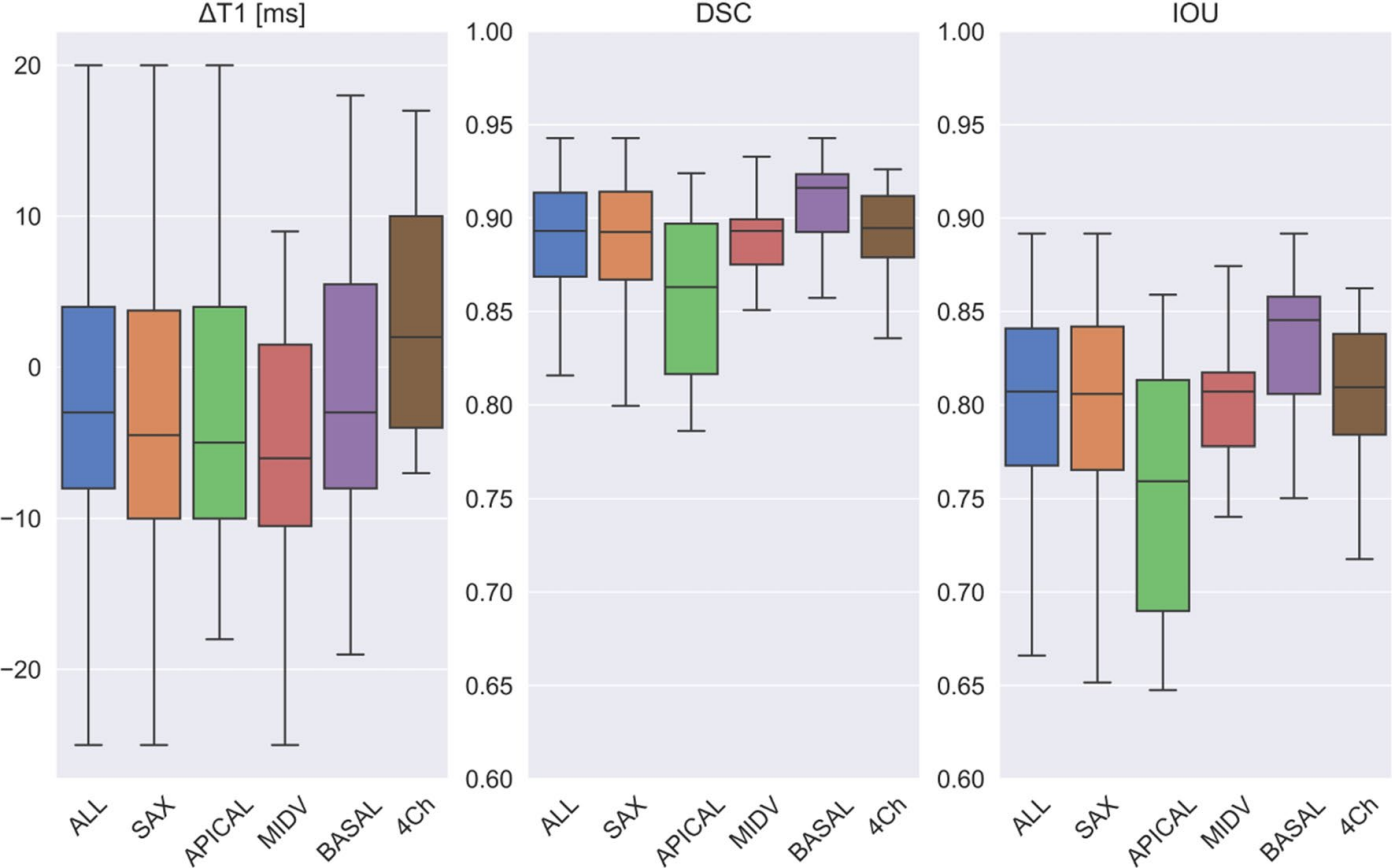
Model statistics [EP35DSCRE]: Boxplot results for all measured metrics (ΔT1, DSC and IOU) in the test cohort between model predictions and ground truth mask, shown for all views, short-axis views only and separately for each view (apical, midventricular, basal, and 4Ch).

Figure 6 shows the performance of the model [EP35DSCRE] for all views. The IQR was ΔT1 ≤ 15 ms for all views with the deviations being predominantly negative in for the SAX views and predominantly positive in for the 4Ch views. Segmentation was equally accurate for long-axis views (mean error ΔT1: 6.77 ± 8.3 ms, mean DSC: 0.89 ± 0.03, mean IOU: 0.81 ± 0.04) as for short-axis images (mean error ΔT1: 11.6 ± 19.7 ms, mean DSC: 0.88 ± 0.08, mean IOU: 0.79 ± 0.1). On the short axis, performance for the apical slices was inferior (mean error ΔT1: 15.9 ± 28.2 ms, mean DSC: 0.84 ± 0.11, mean IOU: 0.73 ± 0.1) compared to basal (mean error ΔT1: 7.9 ± 9.7 ms, mean DSC: 0.91 ± 0.02, mean IOU: 0.84 ± 0.04) and mid-ventricular (mean error ΔT1: 9.4 ± 12.3 ms, mean DSC: 0.89 ± 0.02, mean IOU: 0.81 ± 0.04) slices.

**Figure 7:**
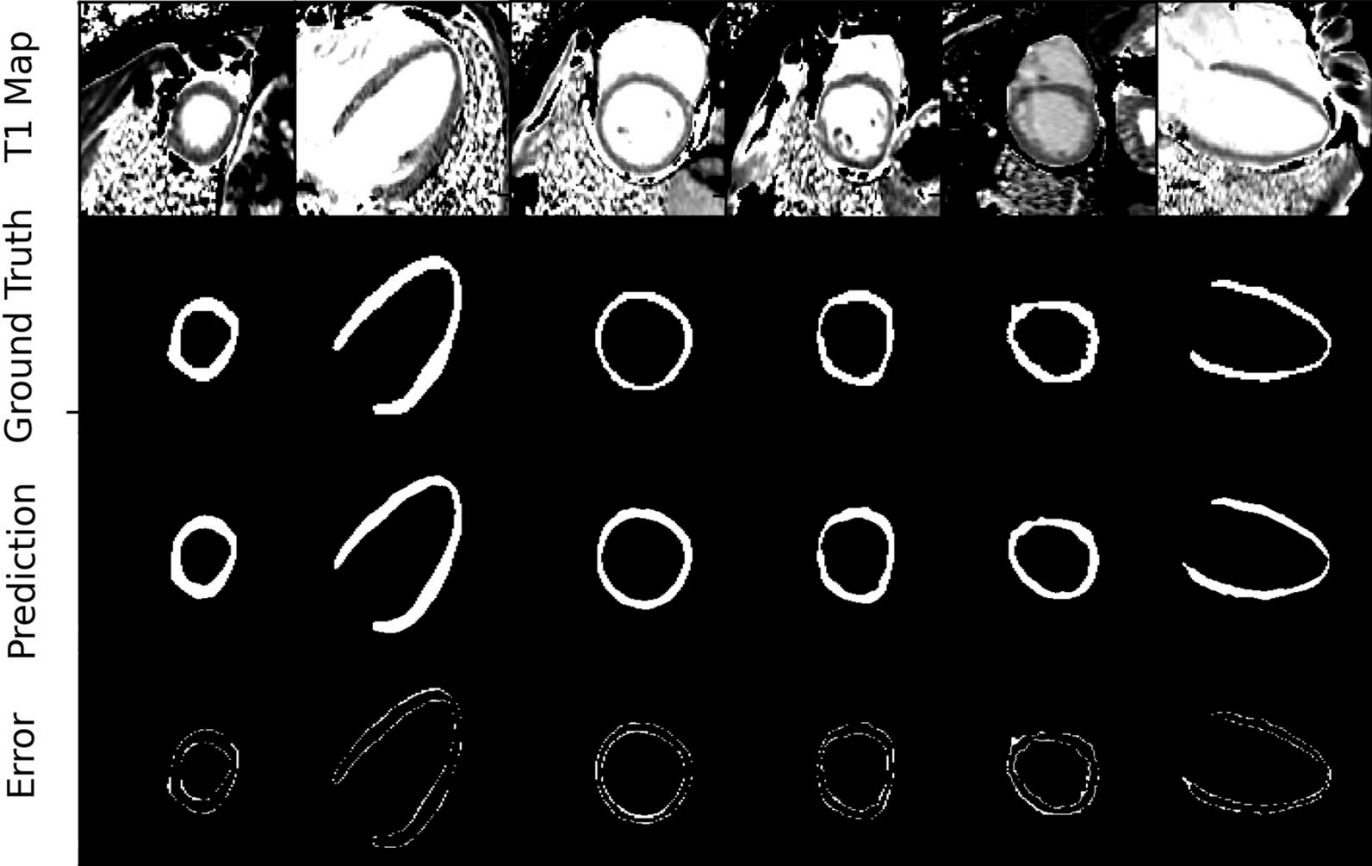
Best model [EP35DSCRE] predictions and ground truth masks: Example of comparison between predicted and ground truth binary masks and segmentation error with a zoom factor of 2.3.

**Figure 8:**
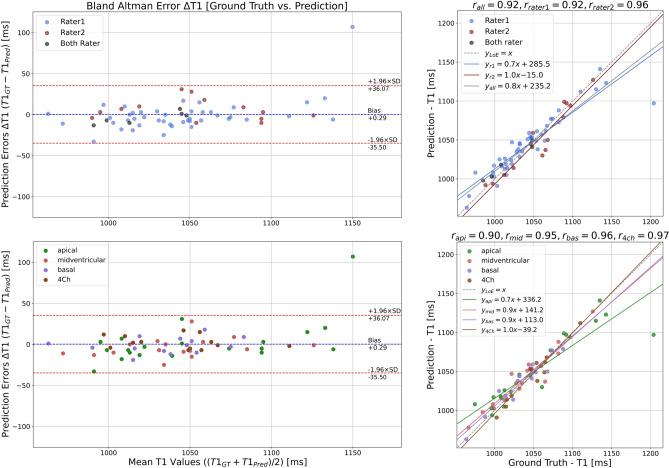
View- and Rater-clustered (top/bottom) model errors: The clustered Bland–Altman analyzes for ΔT1 show all deviations for the test cohort divided for both raters (top) and divided by layer (apical, mid-ventricular, basal, and 4Ch) below. The linear dependencies between ground truth and predictions (right) show all linear fits in comparison to the line of equality (y_LoE = x), which represents an optimal reliability, for the test cohort, split for both raters (top) and split by layer (apical, midventricular, basal and 4Ch) below. This clarifies that the spread of measurement uncertainty becomes larger for smaller regions (apical and basal) and the model fits best for both raters.

Examples of predicted and ground truth masks are shown in Fig. 7. On Bland–Altman analysis, limits of agreement between model results and ground truth were from -35.5 to + 36.1 ms (Fig. 8*).* In 92.1% of cases, the error ΔT1 was within ± 20 ms.

### Model performance in the context of inter-rater variability

We further aimed to quantitatively compare the variability between DL-based model vs. human evaluation to the inter-rater variability between two human assessors. The limits of agreement between model results and ground truth were from -35.5 to + 36.1 ms (Fig. 8), this was superior to the agreement between two human assessors (-34.7 to + 59.1 ms). Furthermore, the model’s mean prediction bias (0.29 ms) was significantly smaller than the mean interrater bias (-12.2 ms).

**Figure 9:**
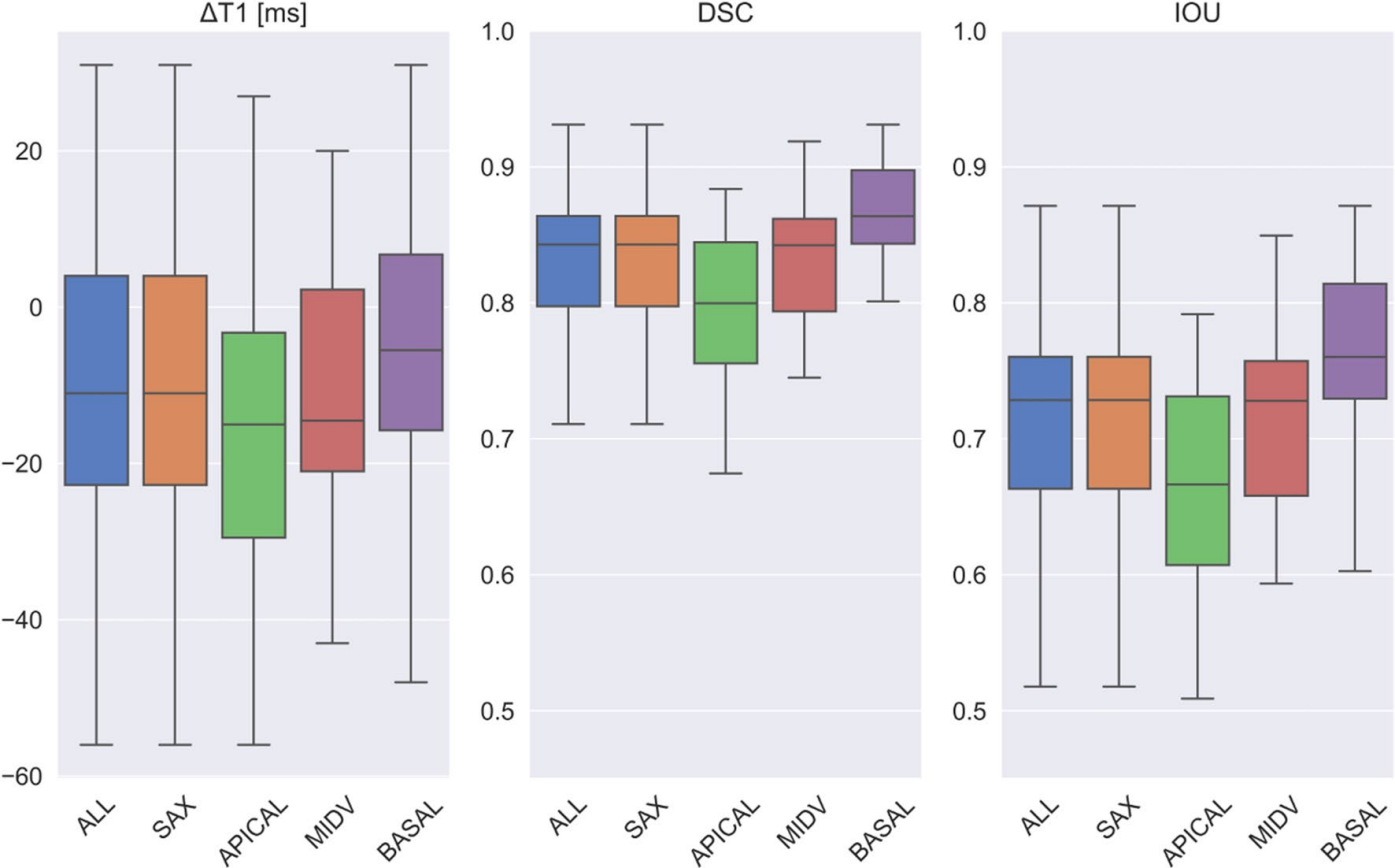
Interrater statistics: Boxplot results for 90 SAX slices for all measured metrics (ΔT1, DSC and IOU) in the test cohort between both rater ground truth mask, shown for all short-axis views and separately for each view (apical, midventricular and basal).

In a more detailed analysis of interrater variability, we further found that the DL-based model’s segmentation accuracy correlated with inter-reader agreement: Both model accuracy and inter-reader agreement were inferior for apical slices and superior for basal and mid-ventricular slices (compare Fig. 6 and Fig. 9). For ΔT1, we found that the error of model vs. human was significantly smaller than the difference human vs. human (interrater variability) specifically for apical slices (p = 0.013), while this was not significant for mid-ventricular and basal slices (Table 3). Geometrical segmentation accuracy of model vs. human was significantly higher than the agreement between two human segmentations for all short-axis slice positions.

**Table 3:** Metrics of best model and interrater comparison.

		ΔT1			DSC			IOU		
		Median	Range	p	Median	Range	p	Median	Range	p
SAX	Prediction	− 4.5	− 33—107	**0.006**	0.892	0.43—0.94	** < 0.001**	0.806	0.27—0.89	** < 0.001**
	Interrater	− 11.0	− 109—31		0.842	0.65—0.93		0.729	0.48—0.87	
API	Prediction	− 5.0	− 33—107	**0.013**	0.863	0.43—0.92	**0.003**	0.759	0.27—0.86	**0.003**
	Interrater	− 15.0	− 89—27		0.799	0.67—0.88		0.666	0.51—0.79	
MID	Prediction	− 6.0	− 25—28	0.11	0.893	0.85—0.93	** < 0.001**	0.807	0.74—0.87	** < 0.001**
	Interrater	− 14.5	− 109—20		0.842	0.65—0.92		0.728	0.48—0.85	
BAS	Prediction	− 3.0	− 19—18	0.56	0.916	0.86—0.94	** < 0.001**	0.845	0.75—0.89	** < 0.001**
	Interrater	− 5.5	− 48—31		0.864	0.75—0.93		0.760	0.60—0.87	

All metrics from best model were compared with interrater metrics for SAX slices.

P-values are from Mann–Whitney U-Test.

Significant values are in bold.

## Discussion

### Related work

While there is abundant literature on fully automated segmentation in cardiac MRI, the majority of these studies have focused on cine sequences (aimed at visualizing and quantifying ventricular and atrial function). In contrast, mapping is performed to quantitatively evaluate biophysical properties of the myocardium, which can hint at underlying cardiac diseases. Only few studies have investigated automated segmentation of T1 maps. In one retrospective study a convolutional neural network was trained to segment myocardial T1 and T2 maps using an edge probability estimation approach ^18^. This previous study was based on a different sequence type (mSASHA – with T1 and T2 maps acquired simultaneously), while our study is based on the more commonly used Modified Look-Locker inversion recovery (MOLLI) sequence. In contrast to our analysis, Howard and colleagues included only short-axis maps and chose a segment-analysis based on the AHA model of 16 myocardial segments. Like in our study, the agreement in segmentation between the neural network and human experts (DSC: 0.82—0.86) was comparable to the agreement between two human experts (DSC: 0.84).

Bhatt and colleagues employed synthetic contrast augmentation in the development and testing of a deep learning-based algorithm for segmentation of T1 maps^14^. Their method demonstrated high accuracy in myocardial segmentation (DSC: 0.81) and T1 calculation (correlation coefficient R = 0.87). Although they were able to show that this approach provides a small increase in accuracy, synthetic contrast augmentation substantially increases the complexity of the network architecture and computational demands. We therefore aimed to develop an algorithm which works on “raw” T1 maps without requiring artificial contrast augmentation.

Several other previous studies have developed algorithms for automated T1 map segmentation with notable difference in network architecture and the underlying imaging dataset^15,16^. Two independent studies developed T1 map segmentation pipelines that combine both segmentation and quality-control steps^13,17^. In this approach, not one but several models and combined models are employed to segment the myocardium on each dataset. From the resulting segmentations, a quality scoring model then selects the optimal segmentation based on predicted segmentation accuracy. In a similar approach, Puyol-Antón and colleagues developed a neural network with Bayesian inference for T1 map segmentation which additionally provides information on segmentation uncertainty^19^. This could be an interesting approach for clinical routine in which the human reader is alerted to review areas of uncertainty after a fully automated segmentation has been carried out.

Our study is in good agreement with the existing literature as we have shown that fully automated segmentation of the left ventricular myocardium is feasible using CNNs with a U-net architecture with an accuracy comparable to the agreement between two human experts. All previous studies on fully automated deep learning-based segmentation of T1 maps were limited to maps acquired in the short axis of the left ventricle, in which the left ventricular myocardium has the approximate shape of a closed circle. Our study is unique in including both short-axis and long-axis (4 chamber view) T1 maps. On long axis views, the left ventricular myocardium forms a U-shape. Thus, including both short- and long-axis views increases the complexity of the segmentation task. Interestingly, we found that segmentation performance was as good for long-axis views (mean DSC: 0.89 ± 0.03) as for short-axis images (0.88 ± 0.08). Accuracy in T1 quantification was slightly superior for long-axis views than for short-axis images (mean error ΔT1: 6.77 ± 8.3 vs 11.6 ± 19.7 ms), largely driven by segmentation inaccuracies in the apical short-axis slices. In apical slices with very thin myocardium, even small deviations in segmentation can lead to relatively large differences in T1 times (Supplementary Material, Fig. S1).

### Implications for practice

In contemporary clinical practice, T1 maps are often analyzed by manually drawing a region of interest in the interventricular septum. This introduces substantial inter-observer variability. Global analysis of myocardial T1 values across the myocardium has been shown to be non-inferior to placing a ROI in the septum, but requires contouring of the myocardium, which is time-consuming and again prone to inter-observer variability^20^. There is considerable variability in the clinical use of T1 mapping in cardiac MRI. Most commonly, one or three short-axis slices are acquired although some centers acquire long-axis slices or both. Here, we propose a deep learning-based algorithm, which can segment the myocardium on T1 maps and provide mean quantitative results for myocardial T1 relaxation time without human interaction. The algorithm is intended for integration into the clinical workflow of cardiovascular imaging specialists. Integration of the algorithm either within the scanner software or into post-processing software with bidirectional access to the Picture Archiving and Communication System (PACS) could ensure that results of T1 analysis can be directly sent to PACS and are ready before physicians even start reading the study.

### Study limitations

Our study was limited to the global quantitative analysis of T1 maps, i.e. the average T1 values across segmented myocardium. This approach is suitable for the diagnosis of pathologies with diffuse changes of the myocardium, such as cardiac amyloidosis, Fabry’s disease and diffuse fibrosis. Other pathologies, most notably myocardial infarction and myocarditis, often manifest as focal changes in T1 values and may not lead to significant changes in mean T1. Therefore, even with automated analysis of mean T1 values, visual analysis of T1 maps for focal changes will still be required. All maps were derived from one 1.5 T MRI scanner of one vendor. Further work is required to confirm the performance of the algorithm for fully automated segmentation of T1 maps from other vendors or acquired at different field strength. Similarly, our study was limited to native T1 mapping. We chose to investigate the influence of activation function and metric on model performance. Other factors such as learning rate, the number of layers, the number of filters for each layer, and loss functions may also influence model performance and were not systematically assessed in our study. The performance was evaluated on a test cohort of previously unseen images from our center. External validation with data from other vendors and centers was beyond the scope of this study. Without further validation it is not clear if the algorithm will perform equally well for T2 maps, post-contrast T1 or extracellular volume (ECV) maps. Since this was a retrospective study, we did not directly show that fully automated in-line analysis of the myocardial T1 maps is feasible in clinical routine. This will require the algorithm to be integrated into either the MRI console itself or into post-processing software connect to the hospital PACS.

## Conclusion

The highest-performing algorithm developed in our study can segment the myocardium on T1 maps both in long-axis and short-axis orientation with very high accuracy. This allows for fully automated quantitative analysis of myocardial T1 maps.

### Supplementary Information

Supplementary Figure S1.

## Data Availability

All data are available upon request from the corresponding author. The software source code is freely available on Github (https://github.com/voxelacrobat/MRI-T1-Mapping-Segmentation , Version 1.0).

## Abbreviations

4Ch: Four-chamber view
AHA: American heart association
API: Apical
BAS: Basal
CNN: Convolutional neural network
DSC: Sørensen-dice-coefficient/dice score
DICOM: Digital imaging and communications in medicine
ECG: Electrocardiogram
IOU: Intersection over union (Jaccard Coefficient)
IQR: Interquartile range
IR: Inversion-recovery
LAX: Long axis
LeakyReLU: Leaky rectified linear unit
MID: Mid-ventricular
MOLLI: MOdified Look-Locker Inversion recovery
MRI: Magnetic resonance imaging
mSASHA: Modified saturation recovery single-shot acquisition
SAX: Short axis
ReLU: Rectified linear unit

## References

[1] World Health Organisation. Cardiovascular diseases (CVDs) fact sheet. https://www.who.int/en/news-room/fact-sheets/detail/cardiovascular-diseases-(cvds). Accessed 7 8 (2023).

[2] Messroghli, D. R. *et al.* Clinical recommendations for cardiovascular magnetic resonance mapping of T1, T2, T2* and extracellular volume: A consensus statement by the Society for Cardiovascular Magnetic Resonance (SCMR) endorsed by the European Association for Cardiovascular Imaging (EACVI). *J. Cardiovasc. Magn. Reson.* **19**, 75. 10.1186/s12968-017-0389-8 (2017). 28992817 10.1186/s12968-017-0389-8 PMC5633041

[3] Puntmann, V. O., Peker, E., Chandrashekhar, Y. & Nagel, E. T1 Mapping in characterizing myocardial disease: A comprehensive review. *Circ. Res.* **119**, 277–299. 10.1161/CIRCRESAHA.116.307974 (2016). 27390332 10.1161/CIRCRESAHA.116.307974

[4] Bai, W. *et al.* Automated cardiovascular magnetic resonance image analysis with fully convolutional networks. *J. Cardiovasc. Magn. Reson.* **20**, 65. 10.1186/s12968-018-0471-x (2018). 30217194 10.1186/s12968-018-0471-x PMC6138894

[5] Bernard, O. *et al.* Deep Learning techniques for automatic MRI cardiac multi-structures segmentation and diagnosis: Is the problem solved?. *IEEE. Trans. Med. Imaging.* **37**, 2514–2525. 10.1109/TMI.2018.2837502 (2018). 29994302 10.1109/TMI.2018.2837502

[6] Böttcher, B. *et al.* Fully automated quantification of left ventricular volumes and function in cardiac MRI: clinical evaluation of a deep learning-based algorithm. *Int. J. Cardiovasc. Imaging.* **36**, 2239–2247. 10.1007/s10554-020-01935-0 (2020). 32677023 10.1007/s10554-020-01935-0 PMC7568707

[7] Dangi, S., Linte, C. A. & Yaniv, Z. A distance map regularized CNN for cardiac cine MR image segmentation. *Med. Phys.* **46**, 5637–5651. 10.1002/mp.13853 (2019). 31598971 10.1002/mp.13853 PMC7372294

[8] Ma, Z. *et al.* An iterative multi-path fully convolutional neural network for automatic cardiac segmentation in cine MR images. *Med. Phys.* **46**, 5652–5665. 10.1002/mp.13859 (2019). 31605627 10.1002/mp.13859

[9] Queirós, S. *et al.* Multi-centre validation of an automatic algorithm for fast 4D myocardial segmentation in cine CMR datasets. *Eur. Heart J. Cardiovasc. Imaging.* **17**, 1118–1127. 10.1093/ehjci/jev247 (2016). 26494877 10.1093/ehjci/jev247

[10] Ruijsink, B. *et al.* Fully automated, quality-controlled cardiac analysis from CMR: Validation and large-scale application to characterize cardiac function. *JACC Cardiovasc. Imaging.* **13**, 684–695. 10.1016/j.jcmg.2019.05.030 (2020). 31326477 10.1016/j.jcmg.2019.05.030 PMC7060799

[11] Suinesiaputra, A. *et al.* Fully-automated left ventricular mass and volume MRI analysis in the UK Biobank population cohort: evaluation of initial results. *Int. J. Cardiovasc. Imaging.* **34**, 281–291. 10.1007/s10554-017-1225-9 (2018). 28836039 10.1007/s10554-017-1225-9 PMC5809564

[12] Vigneault, D. M., Xie, W., Ho, C. Y., Bluemke, D. A. & Noble, J. A. Ω-Net (Omega-Net): Fully automatic, multi-view cardiac MR detection, orientation, and segmentation with deep neural networks. *Med. Image Anal.* **48**, 95–106. 10.1016/j.media.2018.05.008 (2018). 29857330 10.1016/j.media.2018.05.008 PMC7571050

[13] Arega, T. W. *et al.* Automatic uncertainty-based quality controlled T1 mapping and ECV analysis from native and post-contrast cardiac T1 mapping images using Bayesian vision transformer. *Med. Image Anal.*10.1016/j.media.2023.102773 (2023). 36827870 10.1016/j.media.2023.102773

[14] Bhatt, N. *et al.* A deep learning segmentation pipeline for cardiac T1 mapping using MRI relaxation-based synthetic contrast augmentation. *Radiol. Artif. Intell.*10.1148/ryai.210294 (2022). 36523641 10.1148/ryai.210294 PMC9745444

[15] Fahmy, A. S., El-Rewaidy, H., Nezafat, M., Nakamori, S. & Nezafat, R. Automated analysis of cardiovascular magnetic resonance myocardial native T1 mapping images using fully convolutional neural networks. *J. Cardiovasc. Magn. Reson.* **21**, 7. 10.1186/s12968-018-0516-1 (2019). 30636630 10.1186/s12968-018-0516-1 PMC6330747

[16] Farrag, N. A., Lochbihler, A., White, J. A. & Ukwatta, E. Evaluation of fully automated myocardial segmentation techniques in native and contrast-enhanced T1-mapping cardiovascular magnetic resonance images using fully convolutional neural networks. *Med. Phys.* **48**, 215–226. 10.1002/mp.14574 (2021). 33131085 10.1002/mp.14574

[17] Hann, E. *et al.* Deep neural network ensemble for on-the-fly quality control-driven segmentation of cardiac MRI T1 mapping. *Med. Image Anal.*10.1016/j.media.2021.102029 (2021). 33831594 10.1016/j.media.2021.102029 PMC8204226

[18] Howard, J. P. *et al.* Automated inline myocardial segmentation of joint t1 and t2 mapping using deep learning. *Radiol. Artif. Intell.*10.1148/ryai.220050 (2023). 36721410 10.1148/ryai.220050 PMC9885378

[19] Puyol-Antón, E. *et al.* Automated quantification of myocardial tissue characteristics from native T1 mapping using neural networks with uncertainty-based quality-control. *J. Cardiovasc. Magn. Reson.* **22**, 60. 10.1186/s12968-020-00650-y (2020). 32814579 10.1186/s12968-020-00650-y PMC7439533

[20] Böttcher, B. *et al.* Global and regional test-retest reproducibility of native T1 and T2 mapping in cardiac magnetic resonance imaging. *J. Magn. Reson. Imaging* **54**, 1763–1772. 10.1002/jmri.27755 (2021). 34075646 10.1002/jmri.27755

[21] Ronneberger, O., Fischer, P. & Brox, T. U-Net: Convolutional Networks for Biomedical Image Segmentation. In *Medical Image Computing and Computer-Assisted Intervention – MICCAI 2015* (eds Navab, N. *et al.*) (Springer International Publishing, 2015).

[22] Shen, T., Huang, F. & Zhang, X. CT medical image segmentation algorithm based on deep learning technology. *Math. Biosci. Eng.* **20**, 10954–10976. 10.3934/mbe.2023485 (2023). 37322967 10.3934/mbe.2023485

[23] Furtado, P. Testing segmentation popular loss and variations in three multiclass medical imaging problems. *J. Imaging*10.3390/jimaging7020016 (2021). 34460615 10.3390/jimaging7020016 PMC8321275

[24] Müller, D., Soto-Rey, I. & Kramer, F. Towards a guideline for evaluation metrics in medical image segmentation. *BMC Res. Notes.* **15**, 210. 10.1186/s13104-022-06096-y (2022). 35725483 10.1186/s13104-022-06096-y PMC9208116

[25] Handels, H. *Medizinische Bildverarbeitung: Bildanalyse, Mustererkennung und Visualisierung für die computergestützte ärztliche Diagnostik und Therapie | Studium* 2nd edn. (Vieweg+Teubner, 2009).

[26] Kingma D.P. & Ba J. Adam: A Method for Stochastic Optimization. [Conference paper at ICLR 2015, accessed on 22 December 2014]. Available online: https://arxiv.org/abs/1412.6980.

