# Supplementary material for: Development and performance evaluation of fully automated deep learning-based models for myocardial segmentation on T1 mapping MRI data
Source: Sci Rep. 2024 Aug 14;14:18895. doi: 10.1038/s41598-024-69529-7 (PMC11324648; doi:10.1038/s41598-024-69529-7)
Supplement: Supplementary file 1 — Supplementary Figure S1. [file 41598_2024_69529_MOESM1_ESM.docx]

SUPPLEMENTARY MATERIAL


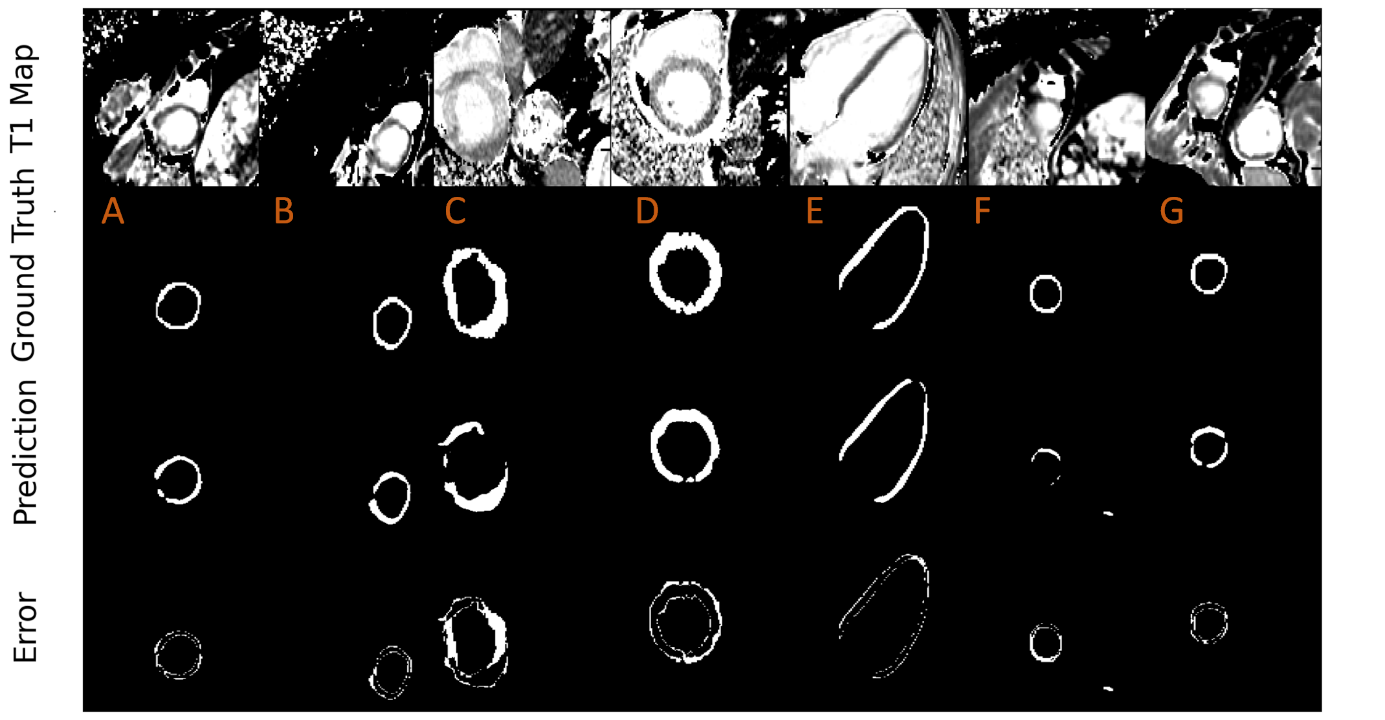


***Fig. S1: Exemplary cases with poor segmentation performance:*** *Original T1 maps, reference masks and segmentation errors are shown here. As the evaluation has already shown, the small apical views are particularly prone to errors, as can be seen in cases A, B, F and G. In case F in particular, the model performed poorly due to reduced image quality caused by motion artifacts.*
